# The Book World of Medicine and Science

**Published:** 1893-10-21

**Authors:** 


### The Book World of Medicine and Science.

Cholera: Where it Comes From and How it is Propagated. By Ernest Hart, Editor of the *British Medical Journal*, Chairman of the English National Health Society.

The editors of the *Chicago Clinical Review* have devoted the whole of their July number to the publication *in extenso* of Mr. Ernest Hart's address on the above subject to the American Medical Association, along with a portrait and short biographical notice of the author. This of itself speaks for the value of the communication, while of the qualifications of the writer it is needless to speak, knowing how closely he has been associated with the investigation of cholera epidemics at home and abroad for the last thirty years. As might be expected, his teaching on the subject goes forth with no uncertain sound. Putting aside for the moment all academic questions of the intimate nature of the disease, he emphatically states at the outset, "That accumulated, and unhappily still accumulating, evidence clearly shows that cholera is a filth disease, carried by dirty people to dirty places, and there spread by the use of dirty water." He then goes on to trace the history of cholera in England, where it was not till the second epidemic in 1849 that Snow had his attention directed to the water supply as the important factor in the spread of the disease. In spite of opposition a third epidemic established his conclusion in the case of the famous Broad Street pump, while twelve years later Mr. Hart himself successfully traced an epidemic to contaminated water. Since that time (1866), though cases have been imported from time to time, cholera has never become epidemic in this country. Mr. Hart then relates the experiences of other countries, devoting a considerable space to the Hamburg epidemic of last year, and in all cases demonstrating that the essential condition for the spread of cholera is the use of specifically polluted water. Tracing it back to its home in the filth-polluted banks of the Ganges, whence it is spread by pilgrims and others, Mr. Hart then shows how much might be done to stamp out cholera in its endemic area by the introduction of a pure water supply. He suggests a system of imperial, provincial, and local sanitary departments for India, which is well deserving of consideration, coming as it does from one so familiar with questions of sanitation. Of the address as a whole, we may say in the words of the *Chicago Clinical Review*, "It is at once scholarly, exhaustive, eminently practical, and timely."

Life with Trans-Siberian Savages. By B. Douglas Howard. (Longmans, Green, and Co.)

It is difficult for the traveller to find a region untrodden by the modern "globe-trotter," but Mr. Howard has succeeded. The last account of the Ainus was written nearly three hundred years ago, and since then their island has been rendered absolutely inaccessible by its conversion into the ultimate penal colony to which double murderers and other distinguished criminals are drafted from Russian and Siberian prisons. Owing to the courtesy of the Governor of this penal settlement the writer enjoyed the opportunity of passing some months in the very centre of the Ainu savages, and of studying their customs and beliefs on the spot. If he has not succeeded in representing them as a very attractive race,

**THE BOOK WORLD OF MEDICINE AND SCIENCE**—*continued.*

he has at any rate produced a valuable record of facts and observations, and parted from his hosts amid demonstrations of affection and regret highly creditable to his character as an explorer. As a "medicine man" his fame seems to have been better founded than that of many amateurs in the art of healing, but the native doctoring is perhaps more interesting, if less efficacious than his own. A certain patient about to be operated upon was suffering from a large abscess consequent on a thrust from a stag. "Just beyond the head of the fire-place, in a space made quite clear for him, sat a rather imposing-looking man, shaving a freshly-gathered stick, which he soon made into an exceptionally large Ineo (idol). This he stuck into the earth floor near the head of the sufferer, around whose head he tied some strips detached from this new Ineo. He then stuffed into his own mouth some sort of herb, as an American cowboy would tobacco. He proceeded to squirt from his mouth with great force the liquid product, first upon the patient, then in every quarter of the hut. He then blew his breath at the same places throughout the hut. This he followed up by using sticks, and fiercely beating the air, as if he were fighting and driving out a small army of invisible enemies. Having sprinkled drops of water and thrown them in all directions, he resumed his place at the head of the hut. . . . Big drops of perspiration poured down his forehead, and his eyes fiercely protruding, seemed staring into infinite distance. In a monotone there came from his stiffened lips sounds as oracular as if from an invisible spirit, every word being slowly spoken and intently followed by the listening crowd, by which the hut was filled to suffocation." The result, it is painful to record, was hardly proportionate to the exertion incurred, but it is satisfactory to learn that the patient was eventually relieved by Mr. Howard with the help of tulip poultices and a sharp penknife. A very interesting account is given of the preparation of the poison with which the Ainus impregnate their arrows. The main ingredients appear to be "a thick extract of the roots of the Aconitum napellus (monkshood), a thinner decoction, or rather infusion, of spider, and thoroughly inspissated fox-gall." The Ainus kill even the animals destined for food with poisoned arrows, taking the precaution of digging out the arrow-head and the heart, which portions are buried as contaminated. "After the carcase in such a case has been thus and by other means thoroughly drained of its blood, the flesh is eaten with impunity." The complete subjection and degradation of the Ainu women, emphasized by their being forbidden to pray or offer any sacrifice to the gods, is a repulsive feature of this strange race. Their sobriety, among many other good qualities, is noted by Mr. Howard with admiration.

